# Brain neuropeptides in central ventilatory and cardiovascular regulation in trout

**DOI:** 10.3389/fendo.2012.00124

**Published:** 2012-10-30

**Authors:** Jean-Claude Le Mével, Frédéric Lancien, Nagi Mimassi, J. Michael Conlon

**Affiliations:** ^1^INSERM UMR 1101, Laboratoire de Traitement de l'Information Médicale, Laboratoire de Neurophysiologie, SFR ScInBioS, Faculté de Médecine et des Sciences de la Santé, Université Européenne de Bretagne, Université de Brest, CHU de BrestBrest, France; ^2^Department of Biochemistry, Faculty of Medicine and Health Sciences, United Arab Emirates UniversityAl Ain, United Arab Emirates

**Keywords:** neuropeptides, brain, ventilatory variables, heart rate, blood pressure, evolution, fish

## Abstract

Many neuropeptides and their G-protein coupled receptors (GPCRs) are present within the brain area involved in ventilatory and cardiovascular regulation but only a few mammalian studies have focused on the integrative physiological actions of neuropeptides on these vital cardio-respiratory regulations. Because both the central neuroanatomical substrates that govern motor ventilatory and cardiovascular output and the primary sequence of regulatory peptides and their receptors have been mostly conserved through evolution, we have developed a trout model to study the central action of native neuropeptides on cardio-ventilatory regulation. In the present review, we summarize the most recent results obtained using this non-mammalian model with a focus on PACAP, VIP, tachykinins, CRF, urotensin-1, CGRP, angiotensin-related peptides, urotensin-II, NPY, and PYY. We propose hypotheses regarding the physiological relevance of the results obtained.

## Introduction

In addition to classical neurotransmitters, numerous brain neuropeptides and their G-protein coupled receptors (GPCRs) have been identified in several cardiovascular and ventilatory nuclei (Fuxe et al., 1986). Considerable data have accumulated in the literature concerning the central cardiovascular actions of these neuropeptides in mammals but much less is known about the roles of central neuropeptides on ventilatory regulation (Niewoehner et al., 1983; Doi and Ramirez, 2008; Pilowsky et al., 2009). Since the central ventilatory system and the cardiovascular system share some nuclei and mutually interact (Niewoehner et al., 1983; Taylor et al., 1999, 2009; Mandel and Schreihofer, 2006; Dampney et al., 2008), it is crucial to determine the integrative role of neuropeptides on these two vital regulatory mechanisms. Fish are aquatic vertebrates that use their gills to breathe, and mammals are vertebrates that breathe using their lungs. Nevertheless, there are important similarities between fish and mammals in the neuroanatomical networks and nervous mechanisms that control the ventilatory and cardiovascular systems (Taylor et al., 1999, 2010a,b; Bolis et al., 2001). In addition, neuropeptides appeared very early during evolution and the primary structures of these peptides and their receptors have been generally well conserved from fish to mammals (Holmgren and Jensen, 2001). Furthermore, in fish as in mammals, the neuropeptidergic systems are frequently present within brain areas involved in cardiovascular and ventilatory functions, including the hypothalamus and the brainstem autonomic nuclei (Batten et al., 1990; Dampney et al., 2005). Consequently, we have developed a trout model to gain insight into the effects of exogenously administered synthetic replicates of endogenous neuropeptides on ventilatory and cardiovascular functions in trout.

In this review, we summarize the neuroanatomical and functional pathways involved in cardio-respiratory control in fish. We describe the trout model and report methods to study the ventilatory and cardiovascular responses to centrally administered neuropeptides. We briefly summarize the available information regarding the primary structures of the fish neuropeptides and the similarities with their mammalian counterparts, the neuroanatomical location of the neuropeptides and their receptors in the fish brains. The neuropeptides investigated in this programme are those whose primary structures are known in the trout and whose neuroananatomical distribution is well characterized. We describe the ventilatory and cardiovascular actions of these neuropeptides following their intracerebroventricular (ICV) injection and we briefly contrast these central effects with their actions following intra-arterial (IA) injection. Finally, we propose hypotheses relating to the potential mechanisms of actions and physiological significance of central neuropeptides in the brain of the trout.

## Neural pathways regulating cardio-respiratory functions in fish

The central control of cardiorespiratory functions in fish has been previously described (Taylor et al., 1999). In fish, the visceral sensory area in the medulla oblongata to which the chemoreceptor and baroreceptor afferent fibers project is homologous to the nucleus tractus solitarius (NTS) of higher vertebrates (Nieuwenhuys and Powels, 1983; Sundin et al., 2003a). The NTS is the site where the first synapse on the chemo- and baro-reflexes takes place. Rhythmic ventilatory movements in fish are generated by a diffuse central pattern generator (CPG) whose activity is modulated by inputs from the peripheral chemoreceptors and also from higher brain centers, including the mesencephalon and the forebrain (Taylor et al., 1999). The CPG controls the activity of trigeminal Vth, facial VIIth, glossopharyngeal IXth, and vagal Xth motor nuclei all of which drive the breathing muscles (Taylor et al., 1999). There is a close association between the neural mechanisms controlling the ventilatory and the cardiovascular systems at the level of the medulla oblongata (Taylor et al., 1999). Furthermore, anatomical and functional links between the hypothalamus and the medullary cardio-respiratory centers in teleosts have been described (Ariëns-Kappers et al., 1936; Hornby and Demski, 1988). Electrical stimulation of hypothalamic sites in the goldfish *Carassius auratus* induces concomitant changes in ventilatory variables and heart rate (HR) (Hornby and Demski, 1988). Within the brainstem, the cardiac vagal pre-ganglionic neurons are located within the dorsal motor nucleus of the vagus (DVN). Some cardiac vagal pre-ganglionic neurons are also present in a more lateral position, probably constituting a primitive nucleus ambiguus. However, little is known regarding the neurotransmitters and/or neuropeptides and their receptors that permit integration of the various inputs at the level of the brainstem to control the final output motor impulses that ultimately govern the ventilatory and cardiovascular variables (Gilmour and Perry, 2007). In the brainstem of the dogfish *Squalus acanthias*, catecholamines regulate the electrical activity of respiratory neurons (Randall and Taylor, 1991). In the channel catfish, *Ictalurus punctatus*, glutamatergic pathways within the caudal part of the NTS are essential for the control of ventilation and studies in the shorthorn sculpin *Myoxocephalus scorpius* reveal that *N*-methyl-D-aspartate (NMDA) receptors mediate ventilatory frequency (VF), while kainate receptors mediate ventilatory amplitude (VA) (Sundin et al., 2003b; Turesson and Sundin, 2003; Turesson et al., 2010). In addition, it was shown that α-amino-3-OH-5-methyl-4-isooxazole-propionic-acid (AMPA) receptors located within the NTS control the parasympathetic activity to the heart and that NMDA and non-NMDA receptors are involved in the hypoxia activated sympathetic hypertension (Turesson et al., 2010). The hearts of teleost fish receive both a cholinergic vagal innervation and an adrenergic sympathetic supply (Taylor et al., 1999). Although humoral catecholamines increase HR after binding to β-adrenoreceptors (Wood and Shelton, 1980), the functional role of the nervous sympathetic system in teleost cardiac control is not clearly recognized (Burnstock, 1969; Taylor et al., 1999). At rest, the teleost heart is under strong inhibitory control mediated by the vagus nerve acting on muscarinic cholinergic receptors on the pacemaker cells (Laurent et al., 1983; Taylor, 1992; Taylor et al., 1999).

## The trout model

For the *in vivo* experiments, we use rainbow trout *Oncorhynchus mykiss* (body wt 240–270 g) of both sexes. The experiments were made on unanesthetized trout under controlled and constant levels of oxygen partial pressure in water (*P*wO2), pH and temperature, maintained at constant levels (*P*wO2 = 20 kPa; *p*H = 7.4−7.6; *T* = 10−11°C). Experimental protocols were approved by the Regional Ethics Committee in Animal Experiments of Brittany, France (registration number: 07).

**Figure 1:**
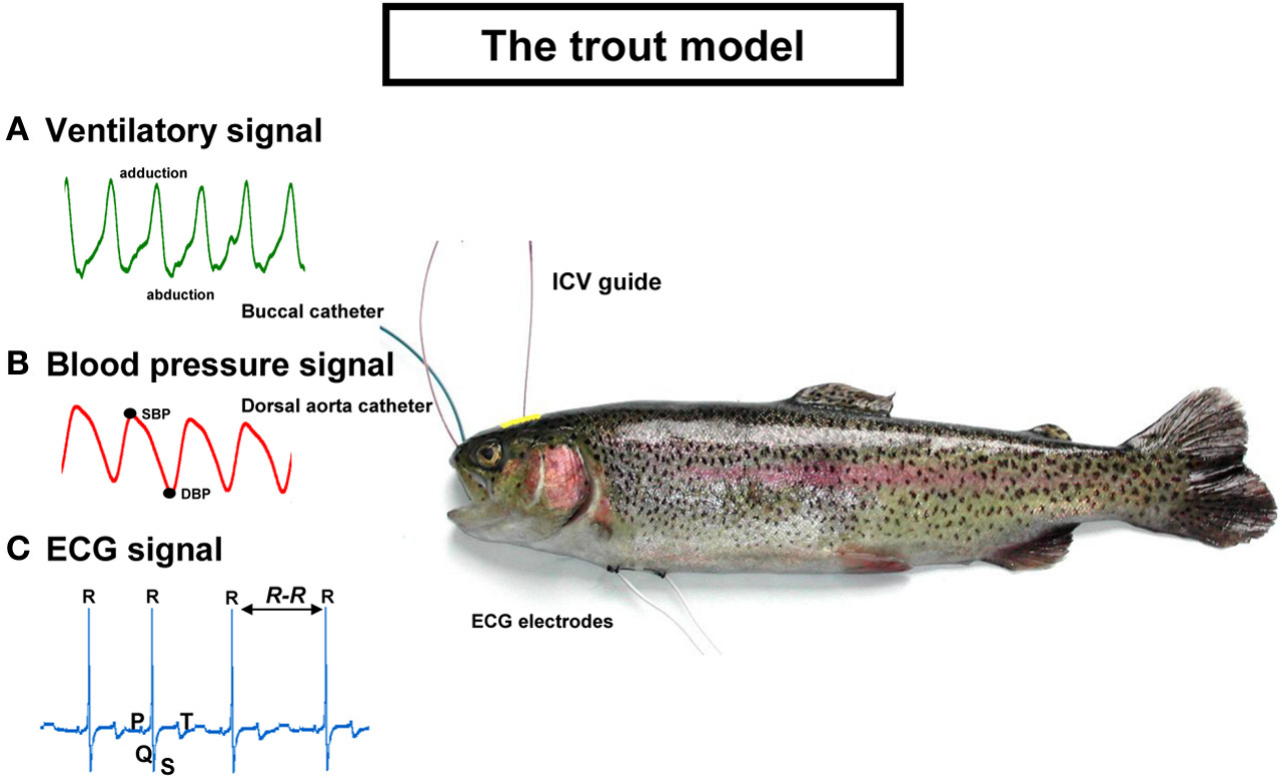
**The trout model used for testing the intracerebroventricular and intra-arterial effects of neuropeptides on **(A)** ventilatory signal; **(B)** blood pressure signal; and **(C)** ECG signal.** SBP, systolic blood pressure; DBP, diastolic blood pressure; ECG, electrocardiographic; ICV, intracerebroventricular. See text for explanations.

An overview of trout equipped with arterial and buccal catheters, electrocardiographic (ECG) leads, and the ICV guide is presented Figure 1. Examples of the recorded signals are also plotted on the figure. The ventilatory and cardiovascular signals are processed off-line with custom-made programs written in LabView 6.1 (Laboratory Virtual Instrument Engineering Workbench, National Instruments).

### The ventilatory signal (Figure 1A)

A flared cannula is inserted into a hole drilled between the nares such that its flared end is resting against the roof of the mouth. This cannula is used to record any changes in buccal ventilatory pressure (Holeton and Randall, 1967). Segments free of any movement artifacts on the ventilatory signal are selected and the VF and VA are determined. VF is calculated from the first harmonic of the power spectrum of the ventilatory signal using the fast Fourier transform (FFT) algorithm. VA is calculated from the difference between the maximal abduction phase and the maximal adduction phase for each of the ventilatory movements. The net effect of the changes in VF and VA on ventilation is determined according to the formula VTOT = VF × VA where VTOT is total ventilation. Thus, the overall ventilatory response is determined by the combined output of the VF and ventilatory stroke volume (by approximation VA).

### The blood pressure signal (Figure 1B)

Catheterization of the dorsal aorta (Soivio et al., 1972) permits the recording of the dorsal aortic blood pressure (P_DA_) and injection of various compounds, including the neuropeptides. Blood is collected via this catheter for routine hematocrit determination and for measurement of the concentration of hormones in plasma. The pulsatile P_DA_ enables the measurement of systolic blood pressure (SBP) and diastolic blood pressure (DBP). Mean P_DA_ is calculated as the arithmetic mean between the SBP and the DBP. The detection of the SBP along the recordings together with the R-R tachogram permits the determination of the cardiac baroreflex response. The baroreflex has been evolutionary conserved from Agnatha (lamprey) to humans (Bagshaw, 1985). The baroreflex in fish, as in humans, is working spontaneously under baseline conditions and also responds to adverse blood pressure changes (Lancien and Le Mével, 2007; Karemaker and Wesseling, 2008). In fish, the baroreflex response is probably used as a mechanism to protect the delicate vasculature of the fish gill against high blood pressure (Sundin and Nilsson, 2002). We evaluate the cardiac baroreflex sensitivity (BRS) using both a time domain method, the sequence method (Bertinieri et al., 1988; Lancien and Le Mével, 2007) and a frequency domain method, the cross spectral analysis technique (Parati et al., 2000; Lancien et al., 2011). In teleost fish (Lancien and Le Mével, 2007; Sandblom and Axelsson, 2011), as in mammals (Bertinieri et al., 1988), the parasympathetic nervous system plays a crucial role in the short term cardiac baroreflex response.

### The electrocardiographic (ECG) signal (Figure 1C)

Two ECG AgCl electrodes are subcutaneously implanted ventrally and longitudinally at the level of the pectoral fins. After amplification, the ECG signal, which is very similar to the human ECG, displays its different waves (P, Q, R, S, T) (Satchell, 1991). The QRS complex is the largest deflection and the R waves are routinely measured to determine HR. The R-R interval of the ECG can be used to plot the tachogram and to quantify the heart rate variability (HRV) using either the FFT algorithm (for review see Task Force of the European Society of Cardiology, and the North American Society of Pacing and Electrophysiology, 1996) or the Poincaré plot (Brennan et al., 2001). HRV reflects modulation on a beat to beat basis of the cardiac sinus node activity by both limbs of the autonomic nervous system. The high frequency component of the HRV in humans reflects respiratory sinus arrhythmia and provides information primarily on the degree of vagal tone on the heart (Médigue et al., 2001). Interestingly, studies of HRV in teleost fish demonstrate that the parasympathetic nervous system is the main, or even the only, contributor to HRV (Altimiras et al., 1995; Le Mével et al., 2002; Grossman and Taylor, 2007). Nevertheless, the physiological significance of HRV in teleost fishes is poorly understood.

### The ICV guide

Fish do not possess large and expanded cerebral hemispheres with a developed neocortex (Nieuwenhuys et al., 1998). Consequently, these animals offer the opportunity to insert directly, under stereomicroscopic guidance, a 25-gauge needle fitted with a PE-10 polyethylene catheter between the two habenular ganglia toward the third ventricle until its tip lies between the two preoptic nuclei (NPO) (Le Mével et al., 2009a). The method is rapid and accurate since no stereotaxic placement is needed. In addition, no post-injection confirmation of the injected site is required. The rationale for this ICV implantation between the two NPO is that neuropeptides, directly or after diffusion through the cerebrospinal fluid, can access sites which are known to be critical to ventilatory and cardiovascular control. In teleost fish, as in mammals, these are the hypothalamus and the brainstem (Hornby and Demski, 1988; Taylor et al., 1999; Dampney et al., 2008).

## Effects of intracerebroventricular injections of neuropeptides

### Pituitary adenylate cyclase-activating polypeptide (PACAP) and vasoactive intestinal peptide (VIP)

PACAP and VIP belong to the secretin-glucagon superfamily of peptides (Sherwood et al., 2000). PACAP is found in two forms, a 38 amino-acid peptide (PACAP-38) and the C-terminally truncated 27 amino-acid peptide (PACAP-27). PACAP and VIP share sequence similarity and, in mammals, these peptides exert their actions by binding to three receptors, PAC1, VPAC1, and VPAC2 (Laburthe et al., 2007). Within the brains of mammals, PACAP and VIP are known to control multiple physiological functions including some cardiovascular and ventilatory processes (Wilson and Cumming, 2008; Vaudry et al., 2009).

PACAP and VIP appeared very early during evolution and the primary structure of these peptides and their receptors have been remarkably well conserved from fish to mammals (Wong et al., 1998; Sherwood et al., 2000; Montpetit et al., 2003). Within the central nervous system (CNS) of teleosts, PACAP- and VIP-like immunoreactivities are localized mainly in neuronal perikarya of the diencephalon at the level of the NPO. Their fibers project not only into the adenohypophysis (Matsuda et al., 1997; Montero et al., 1998; Wong et al., 1998) but also toward many extra hypothalamo-hypophysial areas such as the mesencephalon and the medulla oblongata (Montero et al., 1998). These observations suggest that PACAP and VIP act not only as hypophysiotropic hormones (Montero et al., 1998; Wong et al., 1998) but also as neurotransmitters, and/or neuromodulators. In the goldfish, peripheral PACAP reduces food intake (Matsuda et al., 2006). We also demonstrated that trout PACAP-27 and trout VIP act on the CNS to increase ventilation (Le Mével et al., 2009b) and to reduce the cardiac BRS (Lancien et al., 2011).

After ICV injection, PACAP (25–100 pmol) evokes a dose- and time-dependent elevation of VF and VA. Consequently, the net effect of the peptide is a hyperventilatory response involving a gradual and significant dose-dependent increase in VTOT. The threshold dose for an effect of PACAP on VF is 100 pmol, but a significant effect on VA and VTOT is seen at 50 pmol and this latter effect is observed 15 min after the injection of the peptide. The actions of PACAP on the ventilatory variables are long-lasting since values have not returned to baseline levels by the end of the post-injection period of 25 min. The most pronounced action of PACAP is evoking hyperventilation through an increase in VA rather than VF. For instance, after 50 and 100 pmol PACAP this maximal change in VA, expressed as a percentage of the pre-injection value, reaches about 100 and 200%, respectively, while the elevation of VF is only about 10 and 35% (Le Mével et al., 2009b).

Upon ICV injection, the effects of trout VIP on the ventilatory variables are quite different from those following ICV injection of PACAP. VIP does not produce a significant increase in VF and VA but nonetheless the resultant action of this peptide is a small, transient but significant elevation of VTOT at the highest dose tested. Moreover, statistical analysis of the results obtained following ICV injection indicates that the maximum increase in VF, VA, and VTOT after ICV injection of 100 pmol PACAP relative to the pre-injection values is about 2.5-fold higher than the maximum ventilatory effects of the same dose of VIP.

After ICV injection, only the highest dose of PACAP produces a weak, but significant, sustained increase in P_DA_. However, there is no significant change in HR. ICV injections of VIP do not cause any change in either P_DA_ or HR. The greater action of PACAP on ventilation and blood pressure compared with VIP suggests that PACAP may bind preferentially to PAC1 receptors rather than to VPAC receptors. The lack of HR response to elevation of blood pressure suggests that the cardiac BRS is depressed following central PACAP. Compared with vehicle, ICV injections of PACAP and VIP (25–100 pmol) dose-dependently reduce the cardiac BRS to the same extent with a threshold dose of 50 pmol for a significant effect (Lancien et al., 2011).

In contrast to their ICV effects, IA injections of PACAP and VIP at doses of 25–100 pmol produce no change in the ventilatory variables. Peripherally injected PACAP does not cause any significant change either in P_DA_ or in HR, but bolus peripheral injection of VIP produces a robust dose-dependent and sustained hypertensive response without any change in HR.

### Neuropeptide gamma (NPγ), neurokinin a (NKA) and substance P (SP)

The tachykinins are a family of biologically active peptides that are characterized structurally by the common carboxy-terminal pentapeptide sequence Phe-Xaa-Gly-Leu-Met-NH_2_. This C-terminally amidated sequence is of primary importance for the interaction with the tachykinin receptors (Conlon, 2004). In mammals, SP, NKA, NPγ, and neuropeptide K (NPK) are encoded by the single copy preprotachykinin A gene. Neurokinin B is derived from the preprotachykinin B gene while the preprotachykinin C gene encodes three peptides (hemokinin 1, endokinin C, and endokinin D) with limited structural similarity with SP (for references, see Conlon, 2004). The tachykinins exert their actions by binding to GPCRs that are widely distributed within vascular, endocrine and nervous tissues. SP is the preferential agonist of the NK-1 receptor, NKA along with NPγ and NPK are regarded as endogenous ligands of the NK-2 receptor, and NKB is the preferred agonist of the NK-3 receptor (Patacchini and Maggi, 2004). In mammals, there is strong evidence for the importance of CNS tachykinins in the control of respiration (Gray et al., 1999). In addition, central tachykinins are involved in cardiovascular regulation, neuroendocrine secretion, pain transmission, and in certain behavioral responses (Satake and Kawada, 2006).

Orthologs of the mammalian tachykinins have been isolated and structurally characterized in a wide range of tetrapod and non-tetrapod species (for references, see Conlon, 2004). In particular, SP (Jensen and Conlon, 1992), NKA (Jensen and Conlon, 1992), and NPγ (Jensen et al., 1993) have been purified from tissues of the rainbow trout *O. mykiss*. Neuroanatomical studies have revealed the presence of tachykinin-like immunoreactivity in neuronal cell bodies and fibers throughout the brains of several teleost fish, including the trout (Vecino et al., 1989; Batten et al., 1990; Holmqvist and Ekstrom, 1991; Moons et al., 1992) together with high density of tachykinin binding sites from the hypothalamus to the medulla oblongata (Moons et al., 1992). We recently demonstrated that, after ICV injection, exogenously administered trout tachykinins are differentially implicated in the neuroregulatory control of ventilation in trout (Le Mével et al., 2007).

**Figure 2:**
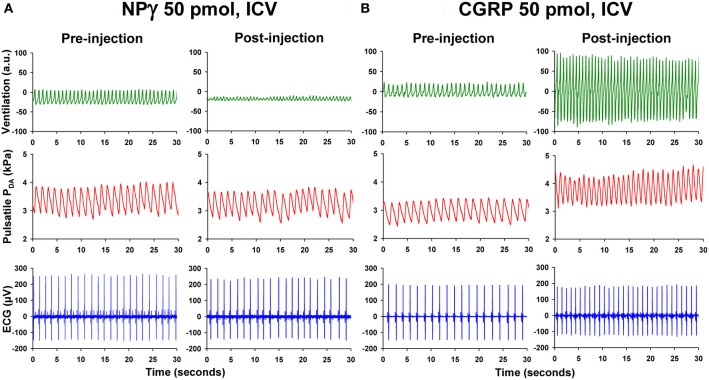
**Recording traces of 30 s duration from two unanesthetized trout illustrating the changes observed in ventilatory movements (ventilation), pulsatile dorsal aortic blood pressure (P_DA_), and electrocardiographic (ECG) signals between the pre-injection period (0–5 min) and the post-injection period (20–25 min) after intracerebroventricular (ICV) injection of (A) 50 pmol trout neuropeptide gamma (NPγ) or (B) 50 pmol trout calcitonin gene-related peptide (CGRP).** Note that ICV injection of NPγ produces an impressive reduction in the ventilatory amplitude but a slight increase in the ventilatory frequency. In contrast, ICV CGRP evokes a potent increase of the ventilation rate and amplitude. Only the ICV injection of CGRP causes a substantial elevation of blood pressure and heart rate.

Compared with ICV injection of vehicle, NPγ (25–100 pmol) evokes a gradual elevation of VF but a progressive dose-dependent reduction of VA. Therefore, the net effect of the peptide is a hypoventilatory response involving a significant decrease in VTOT. The threshold dose for an effect of NPγ on VF, VA, and VTOT is 50 pmol and this is observed 15 min after the injection of the peptide (Figure 2A). Interestingly, in some trout, the ICV injection of 100 pmol NPγ was followed by a dramatic reduction in VA to near the noise level of the recording system for periods of 10–20 s, giving the appearance of an apneic response. All actions of NPγ on the ventilatory variables are of long duration, since parameters do not return to baseline values by the end of the recording period.

In contrast to the action of NPγ, the effects of SP(50–250 pmol) on ventilation are not dose dependent and only the highest dose of SP (250 pmol) produces a significant elevation of VF, a significant reduction of VA, and a resultant significant decrease of VTOT. The changes in these parameters reach significance 10–15 min after ICV injection.

As with SP, the effect of NKA (50–250 pmol) on the ventilatory variables are relatively minor, with only the highest dose (250 pmol) producing a significant decrease in VA and an overall significant fall in VTOT. This action of NKA is of short duration with VA returning rapidly to baseline values.

None of the three tachykinin peptides produce significant changes in mean P_DA_ or HR following ICV injection. Further studies are required to determine whether the central action of NPγ on ventilatory variables in trout involves interaction with a receptor that resembles the mammalian NK-2 receptor more closely than the NK1-receptor.

Because centrally controlled cardiorespiratory coupling contributes to HRV in teleost fish (Grossman and Taylor, 2007), we made the assumption that changes in the VF after central injection of NPγ and SP, but not NKA, also produce changes in HRV (Lancien et al., 2009). Compared to vehicle-injected trout, Poincaré plot analysis of HRV demonstrates that ICV injection of NPγ dose-dependently increases HRV. SP evokes a significant elevation of HRV but only at the highest dose (250 pmol). In contrast, NKA is without any effect on HRV. The physiological significance of HRV in teleost fish is poorly understood. Recent studies favor the hypothesis that HRV may be an important component of the mechanisms optimizing the efficiency of respiratory gas exchange over the counter-current at the gill lamellae (Grossman and Taylor, 2007). Taken together, our data are consistent with a possible selective central action of NPγ on neuronal networks implicated in the control of cardiorespiratory coupling in teleost fish.

IA injections of NPγ, SP or NKA at doses of 50–250 pmol produce no change in any of the ventilatory variables. However, all three tachykinins at their highest dose of 250 pmol cause a significant increase in mean P_DA_ and, except for NPγ, a concomitant and significant fall in HR.

### Corticotropin-releasing factor (CRF) and urotensin-I (U-I)

CRF, a 41-amino acid peptide originally isolated from ovine hypothalamus (Vale et al., 1981), plays a key role in regulating the release of adrenocorticotropic hormone from the pituitary during stress. In mammals, CRF and the CRF-related peptide urocortin 1, an ortholog of the fish U-I (Vaughan et al., 1995; Barsyte et al., 1999), are also known to play a crucial neurotropic role in the CNS in coordinating the autonomic and behavioral responses to stressful situations (Koob and Heinrichs, 1999). In mammals, the actions of the CRF-family peptides are mediated by two types of G-protein-coupled receptors: CRF type 1 receptor (CRF-R1) and CRF type 2 receptor (CRF-R2) (Bale and Vale, 2004). CRF, urocortin-1, and the non-mammalian CRF-related peptides U-I, and sauvagine (SVG) bind with similar affinity to CRF-R1 while CRF has only a low affinity for CRF-R2. U-I, SVG, and the urocortins bind with high affinity to CRF-R2. In mammals including humans, CRF immunoreactivity (Swanson et al., 1983) and CRF receptors (Bale and Vale, 2004) are widely distributed in brain areas involved in the control of cardiovascular regulation and breathing movements. After ICV administration, CRF and urocortin 1 induce marked changes in cardiovascular variables (Parkes et al., 2001) and CRF acts centrally to produce a strong stimulatory effect on ventilatory movements (Bennet et al., 1990).

The CRF family of peptides and their receptors are of ancient origin (Chang and Hsu, 2004). In teleost fish, CRF, U-I, CRF receptors, and CRF binding protein are present not only in neurons of the preoptic region and hypothalamus (Olivereau and Olivereau, 1990) but also in extra-hypothalamic brain regions including the telencephalon and the posterior brain (Batten et al., 1990; Bernier et al., 1999a; Lovejoy and Balment, 1999; Alderman et al., 2008). Taken together, these neuroanatomical findings raise the possibility that CRF and U-I in teleosts also exert extra-hypothalamo-hypophyseal actions and mediate some autonomic and/or behavioral effects within the brain. In fact, physiological data have indicated that, after ICV injection, CRF and U-I are implicated in the autonomic regulation of the cardiovascular system (for review see Le Mével et al., 2006), the control of locomotor activity (Clements et al., 2002) and in the regulation of food intake (for review see Bernier, 2006). Our results demonstrate that CRF and U-I also produce a potent hyperventilatory response when injected centrally in trout (Le Mével et al., 2009a).

After ICV injection, trout CRF (1–10 pmol) evokes both gradual and dose-dependent elevations of VF and VA. The net effect of the peptide is, therefore, a hyperventilatory response involving a significant dose-dependent elevation in VTOT. The minimum dose to elicit a statistically significant response in both ventilatory variables is 5 pmol and this is observed 15 min after the injection of the peptide. In contrast to the sustained action of ICV injection of CRF on VF and VA, a significant stimulatory action of trout U-I (1–10 pmol) on these two variables appears only after ICV injection of the highest dose of peptide tested. VTOT also indicates that the significant hyperventilatory action of U-I is delayed by 10 min (U-I, 5 pmol) and 5 min (U-I, 10 pmol) compared to corresponding doses of CRF. Moreover, the maximum increase in VTOT after ICV injection of CRF relative to the pre-injection value is 2-fold higher than the hyperventilatory effect of U-I during the 25–30 min post-injection period.

At the dose of 5 pmol, only CRF transiently increases P_DA_, but a clear sustained hypertension is observed for the highest dose of 10 pmol of CRF and U-I. ICV injection of either CRF or U-I has no significant effect on HR for all doses tested.

ICV administration of alpha helical CRF_9−41_ (ahCRF_9−41_) alone (50 pmol) does not affect the baseline ventilatory and cardiovascular variables. However, pre-treatment of the trout with this CRF antagonist at a dose ratio of ahCRF_9−41_: CRF of 10:1 delays and significantly reduces (by at least 3-fold) the CRF-induced increase in VF, VA, and VTOT and inhibits CRF-induced elevation in P_DA_. In fish, the pharmacological characteristics of the CRF receptors are quite different from their mammalian counterparts (see above). In catfish, where a third CRF receptor (CRF-R3) has been identified (Arai et al., 2001), CRF-R1 binds CRF, U-I, and SVG with similar affinity, while CRF-R2 preferentially binds SVG. CRF-R3 binds CRF with a 5-fold higher affinity than U-I and SVG (Arai et al., 2001). Pohl et al. (2001) concluded that, in Chum salmon, neither CRF-R1 nor CRF-R2 could discriminate between CRF and U-I. The lack of an intrinsic effect of ahCRF_9−41_ when injected centrally suggests that endogenous CRF and U-I are not involved in the regulation of VA and VF in baseline situations. The fact that this antagonist significantly reduces the central hyperventilatory effects of exogenous CRF is indicative of a selective receptor-mediated hyperventilatory action of CRF in the brain of the trout. However, the type of CRF receptor involved cannot be determined at this time.

After IA injection, CRF and U-I are devoid of any ventilatory or cardiovascular activities except a transient increase in blood pressure at the highest dose of U-I (50 pmol).

### Calcitonin gene-related peptide (CGRP)

The 37-amino- acid peptide CGRP is derived from the tissue-specific splicing of the primary transcript of the calcitonin gene (Amara et al., 1982). CGRP is thus a member of the calcitonin/CGRP peptide family that includes adrenomedullin (AM), adrenomedullin-2 (or intermedin), amylin, and calcitonin receptor-stimulating peptide (Ogoshi et al., 2006; Sawada et al., 2006). CGRP binds to a seven transmembrane G-protein-coupled calcitonin receptor-like receptor that is complexed with one of three receptor activity-modifying proteins (Tam and Brain, 2006). In mammals, CGRP and its receptors are widely distributed throughout the peripheral and central CNS. In the CNS, CGRP acts as a neurotransmitter and/or neuromodulator involved in multiple physiological and behavioral processes including the hypothalamic regulation of feeding (Krahn et al., 1984). In addition, CGRP regulates the local vasodilation of cerebral vessels contributing to the pathophysiology of migraine headache and the peptide modulates pain responses at the level of the spinal cord (Tam and Brain, 2006). Central CGRP also plays a role in the autonomic regulation of the cardiovascular system. In contrast to its hypotensive effect in the periphery, ICV injection of CGRP produces a hypertensive response by activating the sympathetic nerves in rats (Fisher et al., 1983) and CGRP augments the baroreflex controls of renal sympathetic nerve activity and HR in the unanesthetized rabbit (Matsumura et al., 1999).

CGRP has an ancient evolutionary history. In fish, cDNAs encoding for CGRP have been isolated from a number of species (Jansz and Zandberg, 1992; Clark et al., 2002; Ogoshi et al., 2006; Martinez-Alvarez et al., 2008) and CGRP mRNA is expressed in peripheral and central tissues (Clark et al., 2002; Martinez-Alvarez et al., 2008). Moreover, the primary sequence of the peptide has been highly conserved from fish to humans (Shahbazi et al., 1998). As in many cerebral regions, the hypothalamus expresses CGRP mRNA (Martinez-Alvarez et al., 2008) and some CGRP-like immunoreactive fibers represent ascending projections from brainstem areas involved in autonomic functions (Batten and Cambre, 1989; Batten et al., 1990). Interestingly, in the goldfish *Carassius auratus* and in the puffer fish *Fugu rubripes* (Clark et al., 2002) the strongest expression of calcitonin/CGRP transcripts was observed in the posterior brain at the level of autonomic nuclei and spinal cord. In addition, CGRP receptors are present within the brain and heart of the flounder *Paralichthys olivaceus* (Suzuki and Kurokawa, 2000). Collectively these neuroanatomical data support a role for CGRP not only in neuroendocrine function and behavior but also in autonomic and cardiovascular regulation in fish. The anorexigenic action of centrally administered CGRP in the goldfish *Carassius auratus* has been previously described (Martinez-Alvarez et al., 2009). The cardio-ventilatory actions of centrally administered trout CGRP in trout has been recently described (Le Mével et al., 2012).

ICV administration of CGRP (1–50 pmol) evokes a dose- and time-dependent elevation of VF and VA. As a result, the net effect of the peptide is a hyperventilatory response involving a gradual and significant dose-dependent increase in VTOT. The threshold dose for an effect of CGRP on VF is 50 pmol but only 5 pmol for VA (Figure 2B). As for many neuropeptides, the actions of CGRP on these ventilatory variables are long-lasting since values had not returned to baseline levels by the end of the post-injection period of 25 min. This observation suggests that CGRP may act as a long-term hyperventilatory peptide *in vivo*. The most pronounced action of CGRP is evoking hyperventilation through an increase in VA instead of VF. For instance at a dose of 50 pmol, during the 15–20 min post-injection period when VTOT is maximal and increased by 300% from baseline value, the change in VA, expressed as a percentage of pre-injection value, is more than 200% while the elevation of VF is only about 30%.

After ICV injection, CGRP produces a significant dose-dependent and sustained increase in P_DA_ but the increase in HR does not reach the level of statistical significance. The receptor(s) mediating the ventilatory and cardiovascular action of CGRP in trout have not been determined. In eel, the paralogs AM2 and AM5 exhibit different central cardiovascular responses suggesting that they may act through different receptors (Ogoshi et al., 2008).

In contrast to its ICV effects, IA injections of CGRP at doses of 5–50 pmol produce no change in VF, VA, or VTOT. Nonetheless, peripherally injected CGRP causes an overall robust, dose-dependent and sustained hypertensive response without any change in HR. IA injection of the highest dose of CGRP causes at first a rapid but transient decrease in P_DA_ followed by a hypertensive phase that does not return to the pre-injection level until 60 min.

### Angiotensin peptides

Data from mammalian studies have demonstrated that angiotensin II (Ang II) and angiotensin III (Ang III) are the two main effector peptides of the brain renin-angiotensin system (RAS). However, angiotensin IV (Ang IV) and to a lesser extend angiotensin 1–7 (Ang 1–7) are also implicated in various physiological functions, particularly body fluid homeostasis and cardiovascular regulation (Paul et al., 2006; Fyhrquist and Saijonmaa, 2008). Ang II and Ang III bind to angiotensin receptor type 1 (AT1) and type 2 (AT2). Ang IV binds exclusively to angiotensin receptor type 4 (AT4). The type of receptor that mediates the actions of Ang 1–7 is somewhat controversial. Studies on the effects of the RAS on ventilation are limited and only the action of Ang II has been explored in mammals. In both anaesthetized and unanaesthetized dogs (Potter and McCloskey, 1979; Ohtake and Jennings, 1993) and in unanaesthetized rabbits (Potter and McCloskey, 1979) but not in unanaesthetized Sprague-Dawley rats (Walker and Jennings, 1996), Ang II stimulates ventilation through a central mechanism that is independent of baroreceptor or chemoreceptor stimulation (Potter and McCloskey, 1979). In spontaneous hypertensive rats (SHR), but not in normotensive control Wistar-Kyoto rats, intravenous injection of the Ang II receptor antagonist, saralasin, has a depressant action upon ventilation (O'Connor and Jennings, 2001). Because SHR rats exhibit high brain RAS activity compared with normotensive control Wistar-Kyoto rats, the authors speculated that central Ang II is involved in the control of respiration only in SHR rats. However, in anaesthetized Sprague-Dawley rats, intracisternal ANG II provokes a decrease that becomes less when the doses of Ang II are increased (Aguirre et al., 1991) and ICV injection of saralasin reduces respiratory rate and respiratory rate variability in Wistar rats (Olsson et al., 2004). These data indicate that the brain RAS plays a role in the control of ventilation in mammalian species. In humans, Ang II may be implicated in the regulation of the respiratory sensitivity during pregnancy but the mechanism involved in this effect has not been elucidated (Wolfe et al., 1998).

The RAS has an ancient evolutionary history and most of its components are present in lampreys, elasmobranchs, and teleosts (Olson, 1992; Takei et al., 1993; Nishimura, 2001; Rankin et al., 2004; Wong and Takei, 2011). In contrast to the well known peripheral cardiovascular and osmoregulatory hormonal actions of Ang II (Olson, 1992; Le Mével et al., 1993; Bernier et al., 1999b; Takei and Balment, 2009), studies in fish on the central action of Ang II are sparse. Furthermore, two Ang II isoforms [Asn^1^]- and [Asp^1^]-Ang are present in plasma and tissues (Conlon et al., 1996; Wong and Takei, 2012) but the physiological roles of the latter form have only been recently explored (Lancien et al., 2012). Central administration of [Asn^1^]-Ang II into the third or fourth ventricle of the eel *Anguilla japonica* induces drinking (Kozaka et al., 2003) and increases HR and blood pressure (Nobata et al., 2011). This procedure elevates HR and blood pressure but reduces both HRV and the cardiac BRS sensitivity in the trout (Le Mével et al., 1994, 2002, 2008b; Lancien et al., 2004b; Lancien and Le Mével, 2007) In addition, local injection of [Asn^1^]-Ang II within the DVN of the trout potently enhances HR but only weakly increases blood pressure (Pamantung et al., 1997). Taken together, these results demonstrate that in the brains of teleosts, as in mammals, Ang II may act as a neuromodulator or a neurotransmitter involved in key osmoregulatory and cardiovascular regulations. Recently, the cardio-ventilatory actions of exogenously administered [Asn^1^]-Ang II, [Asp^1^]-Ang II, Ang III, Ang IV, and Ang 1–7 within the third ventricle of the trout brain have been described (Lancien et al., 2012). In addition, the angiotensin peptides produced in the brain and circulating in plasma of trout were characterized using a high performance liquid chromatography (HPLC) system that can separate these peptides (Lancien et al., 2012; Wong and Takei, 2012).

After ICV injection (5–50 pmol), [Asn^1^]-Ang II and [Asp^1^]-Ang II gradually elevate VTOT through a selective stimulatory action on VA. However, the hyperventilatory effect of [Asn^1^]-Ang II is 3-fold higher than the effect of [Asp^1^]-Ang II at the 50 pmol dose. Ang III, Ang IV, and Ang 1–7 (25–100 pmol) are without effect on the ventilatory variables. In addition, both Ang II peptides and Ang III dose-dependently increase P_DA_ and HR. These results suggest that the N- and C-terminal amino acid residues of Ang II are important for full effect on the central receptor(s) that mediate(s) hyperventilation and cardiovascular actions. It was previously proposed that in trout, [Asn^1^]-Ang II was the product of angiotensinogen cleavage in plasma but that this peptide is converted to [Asp^1^]-Ang II by plasma asparaginase (Conlon et al., 1996). In eel plasma, asparaginase activity is low and the conversion seems to occur in the tissues such as liver and kidney with angiotensinogen, not Ang II, as substrate (Wong and Takei, 2012). In brain tissue, comparable amounts of [Asn^1^]-Ang II and [Asp^1^]-Ang II were detected (ca. 40 fmol/mg brain tissue) but Ang III was not present, and the amount of Ang IV was about 8-fold lower than the content of the Ang II peptides. In plasma, Ang II peptides were also the major angiotensins (ca. 110 fmol/ml plasma), while significant but lower amounts of Ang III and Ang IV were present. These results demonstrate that the two Ang II peptides are present in trout plasma and brain tissue and suggest that the conversion Asn^1^→ Asp^1^occurs not only in plasma but also in brain. It has been proposed that the teleost AT receptor is an AT1-like receptor (Russell et al., 2001). The demonstration that both Ang II peptides and Ang III elevate P_DA_ and HR while the other angiotensins were without action supports the idea that an AT1-like receptor might also be involved in the central cardiovascular actions of Ang II and Ang III. A novel receptor that binds specifically or with a higher affinity to the Ang II peptides, but not the truncated forms, might mediate the ventilatory effect of the brain RAS in trout.

Within the brain of the trout, [Asn^1^]-Ang II affects not only the mean HR but also the beat to beat change in R-R intervals of the ECG since ICV injection of the peptide reduces HRV (Le Mével et al., 2002) and the cardiac BRS (Lancien and Le Mével, 2007).

None of the angiotensin peptides injected peripherally alter any of the ventilatory variables but the two Ang II isoforms and to a lesser extent Ang III provoke a pressor response. The concomitant decrease in HR following the IA injections of these angiotensins is not significant. In addition, Ang IV and Ang 1–7 are without effect on the cardiovascular variables. Collectively, these results support the view that the N- and C-terminal residues of the Ang II peptides play a role in optimal interaction with the putative cardiovascular angiotensin receptor in trout vascular tissue (Nishimura, 2001).

### Urotensin II (U-II)

U-II is a cyclic neuropeptide that was originally isolated from the caudal neurosecretory system of the teleost fish *Gillichthys mirabilis* on the basis of its smooth muscle-stimulating activity (Pearson et al., 1980; Bern et al., 1985). U-II is widely expressed in peripheral and nervous structures of species from lampreys to mammals including humans (Vaudry et al., 2010). It has now been demonstrated that U-II belongs to a family of structurally related peptides that include U-II and the UII-related peptides (URPs), URP, URP-1, and URP-2. In the teleost lineage, four U-II/URP paralogs are present but only two of these ancestral genes, U-II and URP, are found in tetrapods (Quan et al., 2012). U-II, URP, and URP isoforms exhibit the same cyclic hexapeptide core sequence (Cys-Phe-Trp-Lys-Tyr-Cys) while the N- and C-terminal regions are highly variable (Lihrmann et al., 2006; Conlon, 2008). Studies on UII/URP/URP-2 gene expression in teleosts and tetrapods suggest that U-II, URP, and URP-2 exert different functions (Parmentier et al., 2011). In teleost fish URP, URP-1, and URP-2 mRNA occur both in brain and spinal cord (Parmentier et al., 2008; Nobata et al., 2011) but in the eel, *Anguilla japonica* the U-II gene is exclusively expressed in the urophysis (Nobata et al., 2011). In tetrapods, the U-II gene is expressed primarily in motoneurons of the brainstem and spinal cord (Vaudry et al., 2010). U-II has been identified as a specific natural ligand of the orphan, G-protein-coupled receptor GPR14 (now renamed the UT receptor) in mammals (Vaudry et al., 2010) and in teleost fish (Lu et al., 2006). U-II and URP both activate the UT receptor with the same potency. The cardiovascular effects of centrally administered U-II and URP in trout and eel have been analyzed. In trout and eel, only a relatively high dose of U-II (500 pmol) evokes an increase in P_DA_ with variable action on HR (Le Mével et al., 1996; Nobata et al., 2011). In addition, the central vasopressor action of URP in the eel is equally efficacious but less potent than the action of U-II (Nobata et al., 2011). The brain structures controlling the ventilatory system in trout seem to be more sensitive to the central action of U-II as only a 50 pmol dose of the peptide produces an hyperventilatory response through a significant increase in VF and VA (Lancien et al., 2004a). At this dose, U-II produces a long-lasting increase in locomotor activity (Lancien et al., 2004a). The effects of central URP and URP isoforms on the ventilatory and cardiovascular systems in trout have not yet been determined.

IA injection of U-II and URP in trout and eel evokes an elevation in P_DA_. In both species, the hypertensive effect of U-II is longer-lasting than that of URP (Le Mével et al., 2008a; Nobata et al., 2011). In trout, U-II only provokes a dose-dependent bradycardia (Le Mével et al., 1996, 2008a), while in the eel, U-II and URP significantly increase HR (Nobata et al., 2011). U-II is devoid of ventilatory actions following systemic injection in trout (unpublished observations).

### Neuropeptide tyrosine (NPY) and peptide tyrosine tyrosine (PYY)

NPY and PYY are two members of the pancreatic polypeptide family of regulatory peptides. These two 36-amino acid peptides contain a tyrosine residue at their N- and C-termini. NPY is the most abundant peptide within the CNS of mammals. The peptide and its GPCRs, designated Y_1_, Y_2_, Y_4_, Y_5_, and Y_6_, are widely distributed in nerve terminals throughout the brain (Dumont and Quirion, 2006). NPY-like immunoreactivity has been demonstrated in many noradrenergic and adrenergic neurons of the medulla oblongata (Fuxe et al., 1986). NPY-containing cell bodies are found in the lateral hypothalamus and NPY innervation of the paraventricular nucleus (PVN) of the hypothalamus arises from the medulla oblongata (Dumont and Quirion, 2006). In mammals, including rat, mouse, sheep, pig, rabbit, and pigeon, NPY has many neuroendocrine regulatory effects within the brain including an orexigenic action and regulation of food intake, anxiety, circadian rhythms, and memory. The central actions of NPY on cardiorespiratory functions remain unclear due to the fact that activation of different NPY receptors have opposite effects on cardiorespiratory variables (Fuxe et al., 1983; Scott et al., 1989; Morton et al., 1999). NPY is also present in peripheral organs, notably in blood vessels and heart. PYY is primarily located in endocrine cells of the lower intestine. In mammals, these two peptides are also involved in peripheral vasoregulation (Zukowska-Grojec et al., 1987; Playford et al., 1992).

NPY and PYY are present in both peripheral and brain tissues in fish (Jensen and Conlon, 1992; Danger et al., 1991). Seven NPY receptors subtypes (Y_1_, Y_2_, Y_4_–Y_8_) bind both NPY and PYY in fish (Salaneck et al., 2008). ICV injection of NPY in goldfish increases feeding (Volkoff et al., 2009). In trout, ICV administration of trout NPY and PYY at doses up to 100 pmol does not have any effect on ventilatory and cardiovascular variables (unpublished data). These cardiovascular results are consistent with a previous study demonstrating that ICV injection of human NPY (0.6–0.8 nmol) in trout exerts only a weak hypertensive action without any change in HR (Le Mével et al., 1991). These results obtained in trout suggest that, contrary to the actions of the other peptides mentioned in this review, NPY and PYY do not appear to have an important role in the central cardiorespiratory regulation in trout. In contrast, cod NPY causes vasodilation in the cod celiac artery (Shahbazi et al., 2002). In the elasmobranchs, the unanesthetized *Scyliorinus canicula* (Conlon et al., 1991) and the anesthetized *Heterodontus portjacksoni* (Preston et al., 1998), IA or intravenous injection of relatively high doses of dogfish NPY or PYY significantly increase blood pressure. However in trout, IA injection of trout NPY and PYY at a 100 pmol dose is devoid of significant cardiovascular effects (unpublished data). These differences between the effects of NPY and PYY in elasmobranchs and teleosts can possibly be explained by the different experimental protocols used or that the location of NPY/PYY cardiovascular receptors in the cardiovascular systems of elasmobranchs differs from that in teleost fishes.

## Possible mechanisms of action and physiological significance

In order to produce changes in ventilatory and cardiovascular variables, ICV injections of neuropeptides must access receptors critical for the control of cardio-ventilatory motor neurons. However, the receptor site(s) initiating cellular transduction mechanisms cannot be deduced from the experiments in which the peptides are injected into the third cerebral ventricle. Nevertheless, neuroanatomical prerequisites and some neurophysiological data exist that might explain the ventilatory and cardiovascular responses to ICV neuropeptides. Since the neuropeptides are injected in close proximity to a major neuroendocrine hypothalamic nucleus, the NPO, it is reasonable to assume that these exogenous neuropeptides may mimic the action of the endogenous peptides after release from neurons belonging to this nucleus. These neuropeptides can then activate arginine vasotocin (AVT) and isotocin (IT) preoptic neurons. AVT and IT preoptic neurons project to the neurohypophysis where the two nonapeptides are released into the general circulation. AVT is well known to increase vascular tone and elevate blood pressure *in vivo* (Le Mével et al., 1996; Conklin et al., 1997). In addition to this neuroendocrine pathway, projection from the preoptic neurons could influence brainstem respiratory and cardiovascular neurons including the NTS and the DVN through the neurogenic route by the release of AVT, IT or other neuropeptides or classical neurotransmitters (Batten et al., 1990; Saito et al., 2004). In the goldfish, functional-anatomical studies have demonstrated the existence of a neural pathway from the preoptic area to the DNV controlling concomitantly ventilation and HR (Hornby and Demski, 1988). In mammals, stimulation of the PVN, a nucleus homologous to the teleostean NPO, can influence brainstem and spinal cord respiratory related mechanisms. Vasopressin and oxytocin parvocellular neurons of the PVN project to important respiratory-related regions of the medulla and spinal cord, including the pre-Bõtzinger complex and the phrenic motor nuclei (Mack et al., 2002). The PVN is also part of the central cardiovascular network that controls the rostral ventrolateral medulla (RVLM) (Nunn et al., 2011). Neurons of the RVLM send excitatory projections to the sympathetic pre-ganglionic neurons in the intermediolateral cell column of the spinal cord to increase HR and blood pressure (Dampney et al., 2005; Pilowsky et al., 2009; Nunn et al., 2011). Although in fish the locations of sympathetic pre-vasomotor nuclei within the medulla are unknown, neuropeptides may also act at the medulla oblongata to influence sympathetic outflow to vascular tissue and chromaffin cells increasing blood pressure. In addition, we can speculate about a possible diffusion of the injected neuropeptides within the cerebrospinal fluid toward critical ventilatory and cardiovascular brainstem nuclei. Consistent with this, receptors for some of the aforementioned neuropeptides are also expressed within the hindbrain (Cobb and Brown, 1992; Moons et al., 1992; Lovejoy and Balment, 1999; Lu et al., 2006). However, the pharmacological characterization of these receptors using specific antagonists/agonists is difficult in fish due to the fact that only drugs designed for mammalian receptors are available. We have noted consistently that the effects exerted by the neuropeptides are usually long lasting, a characteristic that is probably related to their slow rate of metabolism. Alternatively, this long lasting effect of neuropeptides may be due to complex intracellular signalling pathways after binding to their metabotropic GPCRs.

We cannot excluded that some neuropeptides when injected at high doses within the periphery may act at central target sites to increase P_DA_, through leakage of the blood brain barrier. However, a direct action of neuropeptides on vasculature is probably the physiological mechanism involved after peripheral injection.

**Figure 3:**
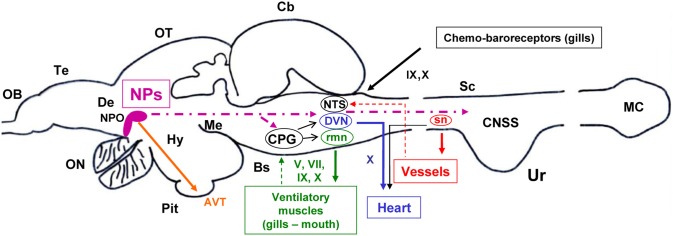
**A model based on a parasagital view of the CNS of the trout depicting the potential central sites and pathways for the effects of intracerebroventricular administered neuropeptides (NPs) on central ventilatory and cardiovascular functions.** Target ventilatory and cardiovascular tissues are also shown. Projecting fibers from preoptic nucleus (NPO) neurons to brainstem ventilatory and cardiovascular nuclei and to spinal sympathetic neurons (sn) are shown with a bold hatched line. Motor outputs from ventilatory and cardiovascular central nuclei to peripheral effectors are shown in continuous line. Feedback information from peripheral tissues to CNS nuclei is shown using thin hatched lines. The sites and pathways described are highly schematic and speculative (see also text in section 2 and 5 for further explanations). Other abbreviations: AVT, arginine-vasotocin; Cb, cerebellum; CPG, central pattern generator; CNSS, caudal neurosecretory system; De, diencephalon; DVN, dorsal motor nucleus of the vagus; Hy, hypothalamus; MC, massa caudalis; Me, mesencephalon; NTS, nucleus tractus solitarius; OB, olfactory bulb; ON, optic nerve; OT, optic tectum; Pit, pituitary gland (hypophysis); rmn, respiratory motor nuclei; Te, telencephalon; Ur, urophysis; VC, valvula cerebelli. V, trigeminal; VII, facial; IX, glossopharyngeal; X, vagal cranial nerves.

Figure 3 gives a summary of the proposed mechanisms for central neuropeptidergic cardio-respiratory regulation in trout.

Table 1 provides a summary of the central ventilatory and cardiovascular actions of neuropeptides in our trout model. However, it remains to be determined whether the observed actions of exogenously administered neuropeptides can be translated into evidence for endogenous regulation of physiological functions. It is probable that a cocktail of neuropeptides within the trout brain is involved in fine control of ventilation, each peptide having a selective action either on VA or VF or on both ventilatory variables. Neuropeptides may be part of the neurochemical systems that are involved in the hypoxic ventilatory response in fish (Porteus et al., 2011). However, as previously stated, their precise implication in the CNS pathways that control the VA and VF during intermittent, repeated or chronic hypoxia is unknown. A balance between the action of hyperventilatory and hypoventilatory peptides may permit the fine control of ventilation so as to maintain homeostasis. In addition, endogenous neuropeptides may regulate cardiovascular function.

**Table 1:** **Summary of the effects of intracerebroventricular injection of neuropeptides on ventilatory and cardiovascular variables in the unanesthetized trout**.

**Neuropeptides (pmol)**		**Ventilatory effects**			**Cardiovascular effects**	
		**VF**	**VA**	**VTOT**	**P**_**DA**_	**HR**
PACAP	(50)	**–**	⬆	⬆	**–**	**–**
	(100)	⬆	⬆	⬆	⬆	**–**
VIP	(50)	**–**	**–**	**–**	**–**	**–**
	(100)	**–**	**–**	⬆	**–**	**–**
NPγ	(50)	⬆	⬇	⬇	**–**	**–**
	(100)	⬆	⬇	⬇	**–**	**–**
SP	(100)	**–**	**–**	**–**	**–**	**–**
	(250)	⬆	⬇	⬇	**–**	**–**
NKA	(100)	**–**	**–**	**–**	**–**	**–**
	(250)	**–**	⬇	⬇	**–**	**–**
CRF	(5)	⬆	⬆	⬆	⬆	**–**
	(10)	⬆	⬆	⬆	⬆	**–**
U-I	(5)	**–**	**–**	**–**	**–**	**–**
	(10)	⬆	⬆	⬆	⬆	**–**
CGRP	(5)	**–**	⬆	⬆	⬆	**–**
	(50)	⬆	⬆	⬆	⬆	**–**
[Asn^1^]-Ang II	(5)	**–**	**–**	**–**	**–**	⬆
	(50)	**–**	⬆	⬆	⬆	⬆
[Asp^1^]-Ang II	(5)	**–**	**–**	**–**	**–**	**–**
	(50)	**–**	⬆	⬆	⬆	⬆
Ang III	(50)	**–**	**–**	**–**	⬆	⬆
	(100)	**–**	**–**	**–**	⬆	⬆
Ang IV	(100)	**–**	**–**	**–**	**–**	**–**
Ang 1–7	(100)	**–**	**–**	**–**	**–**	**–**
U II	(5)	**–**	**–**	**–**	**–**	**–**
	(50)	⬆	⬆	⬆	**–**	⬆
NPY	(100)	**–**	**–**	**–**	**–**	**–**
PYY	(100)	**–**	**–**	**–**	**–**	**–**

PACAP, pituitary adenylate cyclase-activating polypeptide; VIP, vasoactive intestinal peptide; NPγ, neuropeptide gamma; SP, substance P; NKA, neurokinin A; CRF, corticotropin-releasing factor; U-I, urotensin-I; CGRP, calcitonin gene-related peptide; [Asn^1^]-Ang II, [Asn^1^]-angiotensin II; [Asp^1^]-Ang II, [Asp^1^]-angiotensin II; Ang III, angiotensin III; Ang IV, angiotensin IV; Ang 1–7, angiotensin 1–7; U-II, urotensin-II; NPY, neuropeptide Y; PYY, peptide YY. **–**, no effect; ⬆, increase; ⬇, decrease; symbol in bold reflects a higher effect.

The circumstances leading to the release of endogenous neuropeptides into the synaptic cleft to control the ventilatory and cardiovascular autonomic nuclei during adverse metabolic or environmental situations remain to be delineated. It may be hypothesized that the central neuropeptidergic regulation of cardio-respiratory functions may be critical for proper uptake of oxygen from the aquatic environment and distribution of oxygen to tissues during hypoxic stress for example. Hypoxic stress is known to induce an hyperventilatory response in the rainbow trout through a selective action on VA (Gilmour and Perry, 2007) and environmental hypoxia increases the expression of CRF, UI, and CRF-binding protein genes within the NPO (Bernier and Craig, 2005).

## Conclusion and perspectives

Besides conservation of the amino acid sequence of neuropeptides during evolution, our physiological results obtained with unanesthetized trout also give support for a strong conservation of cardiovascular and ventilatory functions throughout the vertebrate classes. Determination of the central ventilatory and cardiovascular actions of these neuropeptides in our trout model suggests that these neuropeptides act as neuromodulators and/or neurotransmitters. In addition, neuropeptides acting as local peptides or hormones may be involved in peripheral cardiovascular regulation. We hope that our comparative physiological studies provide new insights into evolution of the basic neuroregulatory mechanisms that operate in the CNS of vertebrates, including humans, to control these vital cardio-respiratory functions.

### Conflict of interest statement

The authors declare that the research was conducted in the absence of any commercial or financial relationships that could be construed as a potential conflict of interest.

## Abbreviations

BRS: baroreflex sensitivity
CNS: central nervous system
CPG: central pattern generator
DVN: dorsal motor nucleus of the vagus
HR: heart rate
HRV: heart rate variability
IA: intra-arterial
ICV: intracerebroventricular
NPO: preoptic nucleus
P_DA_: dorsal aortic blood pressure
VF: ventilation frequency
VA: ventilation amplitude
VTOT: total ventilation.
